# Habitual Functional Electrical Stimulation Therapy Improves Gait Kinematics and Walking Performance, but Not Patient-Reported Functional Outcomes, of People with Multiple Sclerosis who Present with Foot-Drop

**DOI:** 10.1371/journal.pone.0103368

**Published:** 2014-08-18

**Authors:** Marietta L. van der Linden, Julie E. Hooper, Paula Cowan, Belinda B. Weller, Thomas H. Mercer

**Affiliations:** 1 Rehabilitation Sciences, Queen Margaret University, Musselburgh, United Kingdom; 2 Slateford Physiotherapy Clinic, NHS Lothian, Edinburgh, United Kingdom; 3 Kenilworth Medical Centre, NHS Lanarkshire, Cumbernauld, United Kingdom; 4 Anne Rowling regenerative neurology clinic, Edinburgh, United Kingdom; Charite - Universitätsmedizin Berlin, Germany

## Abstract

**Background:**

People with Multiple Sclerosis (pwMS) often experience a disturbed gait function such as foot-drop. The objective of this pilot study was to investigate the medium term effects of using Functional Electrical Stimulation (FES) to treat foot-drop over a period 12 weeks on gait and patient reported outcomes of pwMS.

**Methods and Findings:**

Nine pwMS aged 35 to 64 (2 males, 7 females) were assessed on four occasions; four weeks before baseline, at baseline and after six weeks and twelve weeks of FES use. Joint kinematics and performance on the 10 meter and 2 minute walk tests (10WT, 2 minWT) were assessed with and without FES. Participants also completed the MS walking Scale (MSWS), MS impact scale (MSIS29), Fatigue Severity Score (FSS) and wore an activity monitor for seven days after each assessment. Compared to unassisted walking, FES resulted in statistically significant improvements in peak dorsiflexion in swing (p = 0.006), 10MWT (p = 0.006) and 2 minWT (p = 0.002). Effect sizes for the training effect, defined as the change from unassisted walking at baseline to that at 12 weeks, indicated improved ankle angle at initial contact (2.6°, 95% CI −1° to 4°, d = 0.78), and a decrease in perceived exertion over the 2 min walking tests (−1.2 points, 95% CI −5.7 to 3.4, d = −0.86). Five participants exceeded the Minimally Detectable Change (MDC) for a training effect on the 10mWT, but only two did so for the 2 minWT. No effects of the use of FES for 12 weeks were found for MSWS, MSIS29, FSS or step count.

**Conclusion:**

Although FES to treat foot-drop appears to offer the potential for a medium term training effect on ankle kinematics and walking speed, this was not reflected in the patient reported outcomes. This observed lack of relationship between objective walking performance and patient reported outcomes warrants further investigation.

**Trial Registration:**

ClinicalTrials.gov NCT01977287

## Introduction

Multiple Sclerosis (MS) is a progressive autoimmune disease of the central nervous system. Although symptom manifestation can vary considerably among individuals, the cluster of symptoms comprising fatigue [1], weakness, posture and movement disturbances is common [2]. Heesen et al [3] reported that gait function was most frequently rated as the most important domain by people with MS (pwMS). A common gait problem even in minimally impaired pwMS is decreased dorsiflexion in the swing phase of the gait cycle, i.e. the phase when the foot is not in contact with the ground [4]–[6]. This means that the toe drags or is close to the ground during the swing phase (i.e. foot-drop), which increases the risk of tripping, stumbling and falling. A recent cohort study [7] reported that 150 people with a confirmed diagnosis of MS reported 675 falls and 3785 near falls over a period of three months, 11% of the falls resulted in injury. First-line treatment for foot-drop is usually physiotherapy or the use of an ankle foot orthotic device (AFO) [8]. However, increasingly, Functional Electrical Stimulation (FES) to the pre-tibial muscles, to aid dorsiflexion in swing, is also prescribed. Although FES for people with a stroke has received considerable attention [8], it is only relatively recently that the effects of FES to treat foot-drop have been investigated in pwMS. Studies including pwMS have indicated a direct orthotic effect, defined as the difference between the conditions without and with the assistance of FES recorded at the same assessment, both in new and established users [6], [9], [13] of FES. Direct orthotic effects included an increase in walking speed both over a shorter distance such as the 10 meter walk test [6], [9], [11]–[13] and longer duration walking tasks such as the 4 minute figure of eight walking test [9] and 5 minute walk test [10]. In a preliminary trial with new users of FES, Scheffler [14] did not find a statistically significant effect of FES for the timed 5 m level walk tests over different surfaces and the Timed Up and Go test, but the performance on the stair ascent and descent test was significantly improved with the assistance of FES. Interestingly, ten out of the11 participants in this study preferred FES over no device and 9 out of the 11 preferred FES over using an AFO. In a qualitative study, Bulley et al [15] reported a similar preference (8 out of the 9) for FES over AFO in stroke patients.

A possible therapeutic or training effect of FES, i.e. an improvement in the person's gait without the assistance of FES over time, as opposed to a direct orthotic effect of FES as assessed within a single session, is addressed in longer term studies. Although a few studies have investigated the medium-term and long-term effects of FES to treat foot-drop in pwMS [9], [11], [12], [16], these studies only reported walking speed in the 10 m walk test or in 3 or 4 minute walking tests and did not assess patient reported walking-related outcomes or measures of participation and fatigue. Only two studies have reported the effect of FES to treat foot-drop on self-reported measures in pwMS. Esnouf et al. [17] reported a significant increase in both the satisfaction and performance components of the Canadian Occupational Performance Measure in the FES group compared to an exercise group after a period of 18 weeks of FES. Recently, Taylor et al [13] found statistically significant improvements in both the psychological and physiological components of the MSIS29 after 6 weeks of using FES for foot-drop. However, to our knowledge, there are no studies addressing the medium term effects of FES on important patient-reported outcomes such as walking performance and fatigue or on daily physical activity in pwMS who have been newly prescribed FES treatment. Furthermore, the mechanisms underpinning possible improvements in walking speed after more prolonged use of FES to treat foot-drop, such as the gait kinematics, have not been fully explored.

Therefore, the aim of this study was to investigate the effects of 12 weeks of FES use on gait kinematics and walking performance (primary outcome measures), patient reported outcomes and habitual physical activity (secondary outcome measures) of pwMS who had been newly prescribed this treatment. We hypothesised that a 12 week period of FES use would shift the gait kinematics, both with and without the assistance of FES, closer towards more normal values which would result in improved walking performance and, in turn, might plausibly be associated with improvement in important patient-reported outcomes of walking performance and impact of MS on daily life. Based on the findings by Taylor et al [11], it was also hypothesized that using FES would reduce the effort of walking and that this would result in a decrease of self-reported fatigue.

## Methods

The protocol for this trial and supporting checklist are available as supporting information; see Checklist S1 and Protocol S1.

### Ethics Statement and trial registration

The study was approved by both Queen Margaret University and National Health Service (NHS) research ethics committees (South-East Scotland Research Ethics Committee, reference number 11-AL-0229, see ‘Approval Letter S1) and the NHS research and development office. In accordance with the declaration of Helsinki, all participants provided written informed consent before taking part in the study. The authors confirm that all ongoing and related trials for this intervention are registered with Clinical Trials.gov (NCT01977287).

### Participants

Participants were recruited through a community NHS (National Health Service) physiotherapy service in Edinburgh, UK. People with a clinical diagnosis of MS as defined using the 2005 McDonald Criteria [18] between the ages of 18 and 75 who were considered by a clinical specialist physiotherapist to be suitable for FES to counter dropped foot were eligible for participation in this study. To assess whether a patient was an appropriate candidate for FES, the physiotherapist carried out a comprehensive physical examination. The examination involved the assessment of active and passive range of movement at the ankle with the hip and knee in a flexed and an extended position which provided an indication of muscle weakness and any muscle shortening. Patients who did not have the strength in the affected leg to bend the hip and knee off the couch and hold the weight of the lower limb against gravity when tested in supine lying were considered unsuitable for FES. Also patients in whom walking distances had become extremely limited and were not community walkers were rarely considered suitable. Muscle tone in the gastrocnemius was assessed by moving the ankle passively and attempting to elicit clonus at the ankle when applying a quick stretch. Patients who did not have a plantargrade range of motion at the ankle and had a fixed deformity were considered to be unsuitable for FES.

Those eligible for participation were provided with a Participant Information Sheet and a study invitation letter. Those agreeing to take part visited the gait analysis laboratory at Queen Margaret University, Edinburgh on four occasions, four weeks before the baseline assessment (no FES) and with and without the assistance of FES at baseline and after 6 and 12 weeks of regular device use, which they commenced after the baseline assessment.

At each visit participants underwent a 3D gait analysis assessment; a 10-metre timed walking test and a two-minute walk test, all with and without FES. All 3D gait analysis assessments were carried out without the assistance of FES first, followed by with FES for each assessment visit. The 10 meter and two minute walk tests were performed without the assistance of FES first, followed by same walk tests with the assistance of FES at the baseline and 12 week assessment. At the 6 week assessment, the 10 meter and two minute walk tests were performed with the assistance of FES first and then without FES.

Participants sat down to recover for 5 minutes between each of the tests. Participants were able to use additional walking aids during testing if required. However, if they commenced testing with a walking aid, all further assessments were conducted using the same aid.

### Walking Performance Tests

Participants were asked to walk parallel to a straight corridor wall for ten metres at their preferred walking speed in accordance with Rossier and Wade [19]. The time taken to walk 10 meters was recorded by a stopwatch. The test was repeated and the average was taken for analysis. Participants were also asked to walk continuously around a 16.5 m elliptical course for two minutes [20]. The distance travelled during the two minutes was recorded. In a recent study within-day reliability for the 10 meter and 2 minute walk tests for community walkers with MS was reported between 1.7–2.7 s and 16–22 m respectively [21].

During the first lap and immediately after the test, participants were asked to rate their rate of perceived exertion (RPE) on the Borg Scale [22] where 6 is ‘no exertion at all’ and 20 ‘maximal exertion’. The difference between the RPE immediately after the test and the RPE in the first lap (dRPE) was used for analysis.

### Gait Analysis

Three dimensional gait analysis was undertaken using a 100 Hz eight infra-red camera Vicon Nexus three dimensional (3D) motion analysis system (Vicon Motion Systems, Oxford, UK). Participants had 14 mm diameter passive reflective sphere makers attached to anatomical landmarks of their lower limbs and the pelvis according to the Vicon Plug-In-Gait manual which is based on the Helen Hays marker system [23]. A static trial was conducted using a Knee Alignment Device (KAD) to derive the orientation of the knee flexion-extension axis. The KADs were removed and standard 14 mm reflective markers were attached over the lateral epicondyle of each femur for the walking trials. Each participant performed six trials of approximately five meters for each condition (with and without FES). For the gait analysis trials only, participants walked barefoot with the FES footswitch taped underneath the heel. Ankle angle at initial contact, peak dorsiflexion in swing, peak knee flexion in swing, hip range of motion over the gait cycle and stride length of the stimulated leg were derived for each trial and then averaged for each participant for analysis.

### Step count

Objective measurement of habitual physical activity was recorded by an ActivPAL activity monitor. After each assessment visit, participants were asked to attach the lightweight monitor to one of their legs (anterior thigh) using adhesive gel stickers (Palstickies) and wear this for a period of seven days before returning it to the researcher. The activity monitor records daily step count and the time spent sitting or lying, standing and walking (‘stepping’).and the sit to stand transitions. Standard Error of Measurement for step count for walking at a speed of 3.2 km/h on a treadmill and for self-selected overground walking for healthy individuals has been reported as 6 and 22 steps respectively [24]. Only step count averaged over at least 5 days was derived for analysis in this report.

### Self-reported measures

At the end of each visit participants were given a questionnaire booklet including the MS Impact Scale-29 [25], the Fatigue Severity Score [26] and the MS walking scale [27] to complete at home and return with the activity monitor in an addressed stamped envelope to the researcher.

The Multiple Sclerosis Impact Scale-29 (MSIS-29) is an instrument measuring the physical (20 items) and psychological (nine items) impact of Multiple Sclerosis. Participants were asked to circle one number which best described the impact of MS on their day-to-day life during the last two weeks. The numbers range from ‘1’ (not at all) to ‘5’ (extremely). The total score was calculated by summing the answers to the 29 questions and hence ranged from 29 to 145. Responsiviness for the MSIS-29 has been reported as good, with effect sizes of 0.82 and 0.66 for the physical and psychological scale respectively [25].

The Fatigue Severity Scale [26] consists of nine statements regarding fatigue during the past week. Participants were asked to circle one number between 1 and 7, a low number indicating the statement is not very appropriate and a high value indicating full agreement with the statement. The average of the numbers selected for each of the nine questions is the final score. A higher score indicates a higher impact of fatigue. Learmonth et al [28] reported acceptable reliability of the FSS over six months (ICC = 0.751).

The MS walking scale (MSWS2v1 [27]) consists of 12 items regarding the perceived walking performance over the last two weeks. Walking limitations are self-reported using five response categories generating a total transformed score ranging from 0 to 100, with lower scores indicate better mobility. Excellent reliability of six months (ICC = 0.927) has been reported for the MSWS12v1 with a SEM of 8 and MDC of 22 points [27].

### Functional Electrical Stimulation

The single channel Odstock Drop Foot Stimulator (ODFS III) or the newer version, the Pace (both Biomedical Engineering and Medical Physics, Salisbury, UK) were used to administer FES. Stimulators were fitted and set-up by the physiotherapist and these settings were not changed before or during the assessments. The intensity of the current amplitude ranged from 20 to 70 mA and was determined by the amplitude required to achieve adequate dorsiflexion of the ankle to achieve foot clearance during the swing phase of the gait as decided by a physiotherapist qualified to fit the ODFSIII and Pace. In the majority of patients, standard set-up was used whereby one square 50×50 mm gel surface electrode (PALS, Platinum Blue, Nidd Valley Medical Ltd, Knaresborough Ltd) was placed over the common peroneal nerve as it passes over the head of the fibula and another over the motor point of the Tibialis Anterior. However, in some patients adjustments were required to the positioning or the polarity of the electrodes to produce the desired effect.

### Data analysis

Using the terminology by Taylor et al [12], the following effects of FES use over 12 weeks were assessed in this study. The training effect on the gait kinematics and walking performance tests was defined as the change in gait kinematics and walking performance without FES at 12 weeks relative to the gait kinematics and walking performance without FES at baseline. The direct orthotic effect is the difference in walking outcomes between walking with and without FES at the same assessment, at baseline and the 6 week and 12 week assessments. Finally, the combined effect of the training and direct orthotic effect is defined as the total orthotic effect, i.e. the change in walking outcomes at 12 weeks with the assistance of FES relative to the walking outcomes at baseline without FES.

A doubly repeated measures ANOVA with two within-subject factors of time (three levels; baseline, 6 weeks post-baseline, 12 weeks post-baseline) and FES condition (two levels; with and without the assistance of FES) was used to check for statistically significant effects on gait kinematics and walking test performance. The effects of longer-term FES use on patient-reported outcomes and step count were explored via repeated measures ANOVA with time (three levels, baseline, 6 weeks post-baseline, 12 weeks post-baseline) serving as the only within subject factor. Equivalent non-parametric tests were used for data not found to be normally distributed. A first order autoregressive correlation structure was used in the repeated measures ANOVA model. Where appropriate, we reported the interaction effects and all main effects (p- values) for the within-subject factors of time and condition. Statistical significance was accepted for p-values<0.05. SPSS v 19 was used for statistical analysis.

Cohen's effect size d for the training effect at 12 weeks and the total orthotic effect were calculated to inform the sample size for future, appropriately powered, larger scale trials. Effect sizes were defined to be medium for values for Cohen's d of more than 0.3 but less than 0.5, good for values of 0.5 and greater but less than 0.8, and large for values of 0.8 and greater [29].

The measurements at the assessment four weeks before baseline and the baseline assessment were used to calculate the Standard Error of Measurement and the Minimally Detectable Change. The standard error of measurement (SEM) was calculated from the standard deviation at baseline and the reliability coefficient: SEM = SD * √(1-r), where r is the reliability coefficient (Cronbach's alpha) and the Minimally Detectable Change (MDC) was derived from the SEM: MDC = 1.96*√2 * SEM. The number of participants exceeding the MDC for both the training effect and total orthotic effect were counted.

## Results

**Figure 1:**
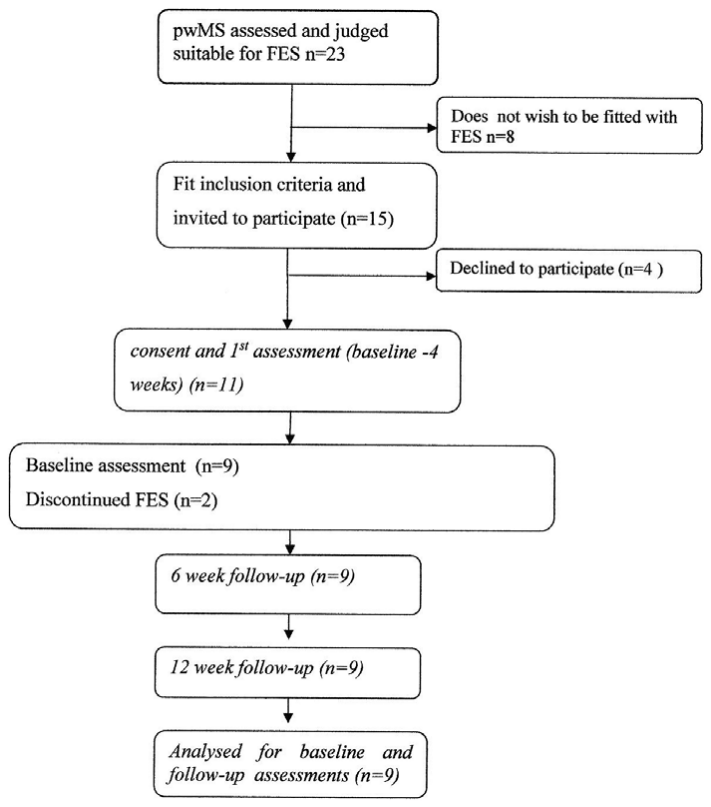
CONSORT diagram.

During the recruitment period (August 2011–April 2013) twenty three people with MS presented with foot-drop and were judged suitable by a specialist physiotherapist for FES to the dorsiflexors. Of those twenty-three, fifteen wished to be fitted with FES and were invited to participate in the study. Eleven gave informed consent and underwent the first assessment (see CONSORT diagram Fig. 1). However, two participants did not return after their first visit as they did not wish to continue with FES therapy, resulting in nine participants being available for follow-up and who were included in the data analysis. Participant characteristics are shown in Table 1. There were no adverse events reported.

**Table 1:** Participant characteristics, means (standard deviation).

Male/female	2/7
Age (years)	53(9) range 35–64
RM/PP/SP	4/4/1
Body Height (m)	1.67(0.09)
Body Mass (kg)	70(14)
Body Mass Index (kg/m^2^)	24.8(2.1)
Walking aid/no walking aid during walking tests	1/8

RM: Relapse-Remitting, PP: primary progressive, SP: secondary progressive.

### Gait kinematics

Gait kinematics are shown in Table 2. Peak dorsiflexion in swing (p = 0.006) and stride length (p = 0.049) were significantly improved in the FES assisted condition compared to no FES, indicating a direct orthotic effect. Over the 12 week period, a significant effect of time, indicating an improvement from baseline was observed for both stride length (p = 0.045) and walking speed (p = 0.046) and just failed to reach significance for ankle angle at Initial Contact (IC) (p = 0.082). Cohen's effect size ‘d’ for a training effect ranged from 0.22 for peak knee flexion in swing to 0.78 for ankle angle at IC, indicating improved, more normal gait kinematics. Compared to the baseline assessment without FES, the following gait kinematics in the trials with FES had good effect sizes indicating improvement at the 12 week assessment (total orthotic effect): ankle angle at IC and peak dorsiflexion in swing (Cohen's d>1.0), stride length and walking speed (d = 0.70 and d = 0.80), knee flexion in swing (d = 0.36) and hip range of motion (d = 0.45).

**Table 2:** Mean (std) values of the gait kinematics in the two conditions (FES switched off and FES switched on) and four assessments and p- values derived from ANOVA for the effects of condition, time and their interaction.

	FES	BL-4 weeks	Baseline	6 weeks	12 weeks	Training effect (d)	Total orthotic effect (d)	p_cond_	p_time_	*p_cond xtime_*
**Angle at IC(°)** *	Off	−4(4)	−4(3)	−3(3)	−1(4)	3(0.78)	3.4(1.01)	0.334	0.082	0.230
	On		−3(3)	−3(1)	0(4)					
**DF swing(°)**	Off	3(3)	3(2)	4(2)	4(4)	1(0.50)	2.9(1.3)	0.006	0.251	0.365
	On		5(2)	5(2)	6(3)					
**Knee flex(°)**	Off	46(9)	46(10)	47(9)	48(9)	2(0.22)	3.6(0.36)	0.135	0.279	0.823
	On		48(9)	48(9)	50(10)					
**Hip rom(°)**	On	36(6.5)	37(8)	41(7)	39(5)	2(0.27)	3.5(0.45)	0.127	0.717	0.003
	Off		41(7)	41(7)	40(6)					
**Stridel(m)**	Off	0.92(0.17)	0.94(0.17)	1.02(0.18)	1.01(0.16)	0.70(0.43)	0.12(0.70)	0.049	0.045	0.092
	On		1.02(0.12)	1.05(0.15)	1.06(0.15)					
**Speed(m/s)**	Off	0.77(0.17)	0.79(0.19)	0.88(0.21)	0.89(0.20)	0.10(0.54)	0.15(0.80)	0.124	0.046	0.385
	On		0.86(0.16)	0.91(0.19)	0.94(0.21)					

Training and Total orthotic effects are given as the difference in outcome between baseline (FES switched off) and 12 weeks, without and with the assistance FES respectively. Cohen' s d is given in brackets.

*a negative sign indicates plantar flexion.

IC = Initial Contact, DF = dorsiflexion, Kneeflex = peak knee flexion in swing, d = Cohen's effect size d.

### Walking performance

Table 3 shows the results of the walking performance tests and gait characteristics with and without FES over the three assessments. Both the 10 meter and the 2 minute walking performance tests showed improved values for the FES assisted condition compared to without FES for all three assessments. A statistically significant direct orthotic effect was found both for the 2 minute walk test (p = 0.002) and the 10 meter walk test (p = 0.006). No statistically significant effect of time was found for any of the walking performance outcomes. Average training effects at 12 weeks were 8.2% for the 10 meter walk test and 4.7% for the 2 minute walk test (Cohen's d<0.29). The difference between the RPE at the end of the 2 minute test compared to the first lap was on average 1.2 points lower at 12 weeks compared to baseline (Cohen's d = −0.86).

**Table 3:** Mean values (std) of the walking performance tests over the four assessments and two conditions, p- values derived from ANOVA for the effects of condition (FES switched off vs. FES switched on), time and their interaction are also given.

	FES	BL-4 weeks	Baseline	6 weeks	12 weeks	Training effect (d)	Total orthotic effect (d)	p _cond_	p _time_	p _cond x time_
**10 m (m/s)**	Off	0.97(0.26)	0.96(0.22)	0.98(0.25)	1.03(0.24)	8.2%(−0.29)	12.1%(−0.41)	0.006	0.545	0.066
	On		1.03(0.19)	1.06(0.24)	1.07(0.23)					
**2 min (m/s)**	Off	0.83(0.12)	0.84(0.20)	0.83(0.13)	0.88(0.22)	4.7%(0.20)	9.8%(0.42)	0.002	0.209.	0.935
	On		0.87(0.18)	0.87(0.22)	0.92(0.25)					
**dRPE**	Off	4.0(1.6)	4.5(1.3)	3.3(2.3)	3.3(1.6)	27%(−0.86)	44%(−0.95)	0.198	0.066	0.377
	On		3.1(1.4)	2.5(1.3)	2.5(1.3)					

Training and Total orthotic effects are given as the percentage improvement between baseline (FES switched off) and 12 weeks, without and with the assistance FES respectively. Cohen' s d is given in brackets.

d = Cohen's effect size d.

The total orthotic effect, the change in walking speed with FES at 12 weeks relative to the walking speed without FES at baseline was 12.1% (Cohen's d = −0.41) for the 10 meter walk test and 9.8% (Cohen's d = 0.42) for 2 minute test. A large effect size (Cohen's d = −0.95) for total orthotic gain was found for the dRPE indicating a lower increase in RPE over 2 minutes in the FES assisted condition after 12 weeks compared to without FES at baseline.

### Self-reported measures and objective physical activity

Table 4 shows the self-reported measures and daily step count over the three assessments. There were no statistically significant effects of time for any of the measures except for the MS walking scale in the FES group. Effect sizes of the change between 12 weeks and baseline were all small (d<0.3). Interestingly, the MS walking scale was significantly lower, i.e. improved at 6 weeks compared to at baseline (p = 0.034) but was back to the baseline level at 12 weeks.

**Table 4:** Mean (std) of the self-reported measures and daily step count, p-values for the effect of time (ANOVA unless otherwise stated) are also included.

	Baseline −4	baseline	6 weeks	12 weeks	Cohen's d	P time
**MSIS29** *	74.0(23.6)	73.0(21.2)	67.3(21.3)	72.3(22.4)	−0.03	0.130
**MSWS** * ¥	75.4(8.2)	70.0(11.8)	61.5(11.7)	69.4(8.9)	−0.04	0.034
**FSS** *	4.8(1.5)	4.8(1.6)	4.9(0.9)	5.1(0.8)	0.22	0.261¥
**Step count**	5353(2872)	5394(1836)	5933(2290)	5758(2406)	0.20	0.363

¥ = Friedman's ANOVA.

* = A higher value indicates a higher impact of MS.

### Patient outcomes exceeding Minimally Detectable Change (MDC)

**Figure 2:**
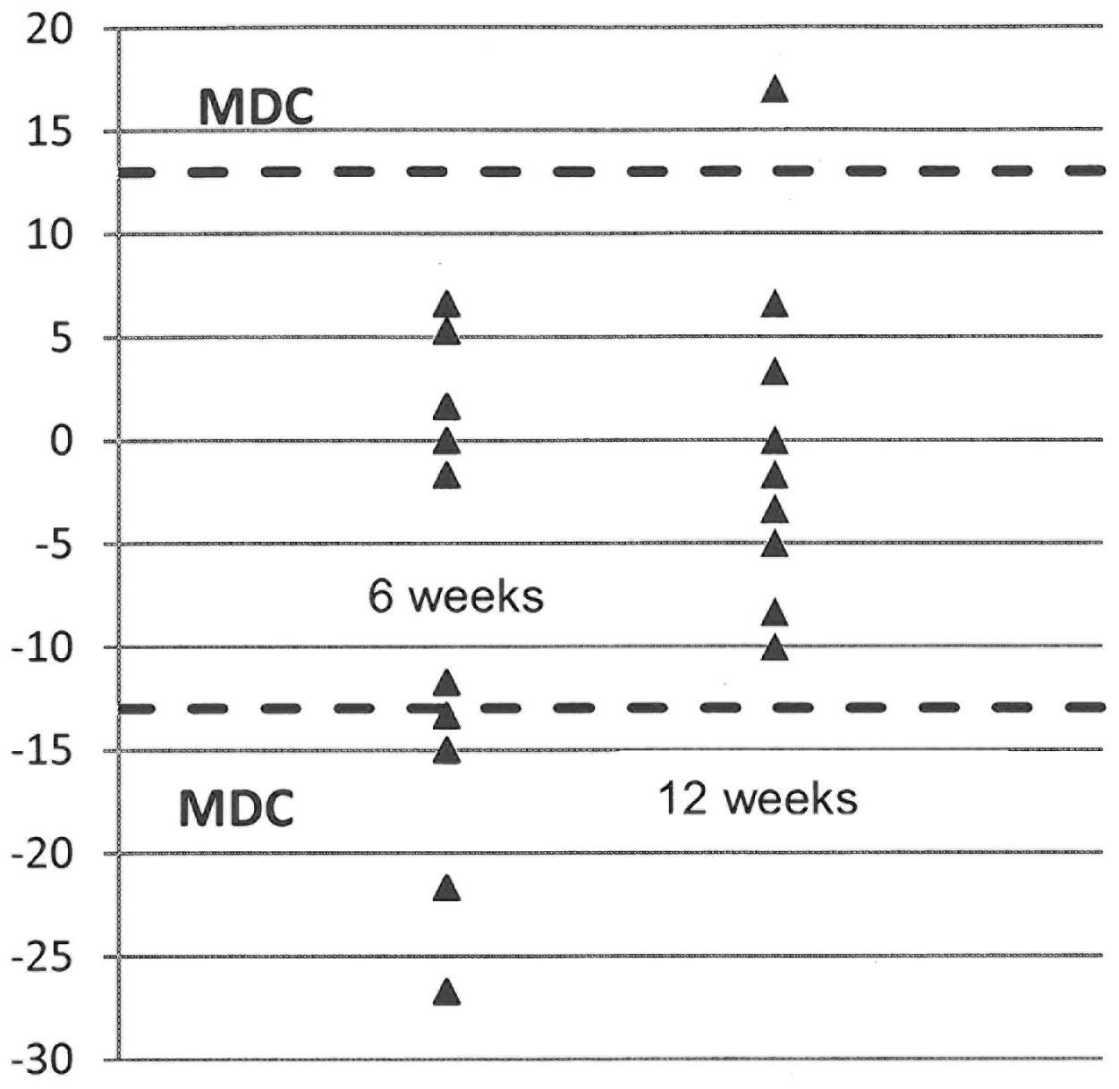
Individual change scores from baseline for MSWS at 6 and 12 weeks. Broken line: Levels of Minimally Detectable Change (MDC) for negative change (indicates improvement) and positive change (indicates deterioration).

The number of participants exceeding the MDC for both the training and total orthotic effect at the 12 week assessment is provided in Table 5. The MDC was derived from participants' baseline and baseline – 4 week scores. Interestingly, although the majority of participants showed improvements exceeding the MDC between 12 weeks and baseline for the 10 meter walk test tests, improvements exceeding the MDC in gait kinematics and self-reported measures were less common. Figure 2 illustrates the individual participant changes scores in relation to the MDC for the MS walking scale.

**Table 5:** Number of participants exceeding the MDC after 12 weeks of FES use for both training effect and total orthotic effect.

	Training effect		Total orthotic effect	
	Better	Worse	Better	Worse
**10 meter walk (MDC = 0.47 s)**	6/9	1/9	6/9	0/9
**2 min walk (MDC = 10.7 m)**	2/8	1/8	5/8	0/8
**Ankle angle at IC (MDC = 4.7°)**	1/9	0/9	2/9	0/9
**Dorsiflexion swing (MDC = 5.3°)**	1/9	1/9	2/9	0/9
**Stride length (MDC = 0.07 m)**	3/9	0/9	4/9	0/9
**MSIS 29 (MDC = 13)**	0/9	1/9		
**MSWS-12 (MDC = 14)**	0/9	1/9		
**FSS (MDC = 0.89)**	3/9	2/9		
**Step count (MDC = 1741)**	0/6	0/6		

## Discussion

The primary aim of this pilot study was to investigate the training and total orthotic effects on gait kinematics and walking performance tests after using FES to treat foot-drop for a period of 12 weeks. We also investigated whether self-reported measures of walking performance, impact of MS on daily living, fatigue and habitual physical activity improve as a result of using FES over a period of 12 weeks.

A statistically significant improvement over time was observed for stride length and walking speed (p<0.05). At 12 weeks the ankle angle at initial contact without the assistance of FES showed a shift towards more dorsiflexion of 2.6 degrees (Cohen's d = 0.78) compared to unassisted gait at baseline, indicating a trend towards a training effect.

Good to excellent effect sizes were found for the total orthotic effect for ankle angle at IC (3.4°, d = 1.0) and dorsiflexion in swing (2.9°, d = 1.3). No previous studies on FES have recorded the total orthotic effects on gait kinematics in people with MS. However, a study with children with CP [30] reported a similar total orthotic gain of 3.5°. Winter [31] showed that a change in joint angle of as little as 2° could significantly alter foot clearance, indicating a possible clinical relevance of the improvements in ankle kinematics found in the current study.

This pilot study showed no statistically significant effect of time for the walking performance tests. Small average training effects on walking performance of 4.7% and 8.1% (Cohen's d<0.3) were found for the 2 minute and 10 meter walking tests respectively. Interestingly, after 12 weeks of FES use, participants showed a trend (Cohen's d>0.8) towards a smaller increase in RPE from the start to end of the two minute walk test with dRPE. This may reflect a lower perception of effort of walking when walking with FES assistance and also a possible carry-over effect when walking without FES after 12 weeks of FES use.

The average training effects on the walking performance tests observed in the current study are similar to those reported in earlier studies. Stein et al [9] observed for their progressive group (all but one consisting of pwMS). who used the WalkAide for three months, improvements of 9.1% for the 4 minute walk test and 5.3% for the 10 meter walk test. A recently published study by Taylor et al [13] assessed the effects of different combinations of FES for foot drop, FES to the hip extensors and home exercises. After 6 weeks of using FES for foot-drop, they reported that participants showed (a non significant) 9.7% increase in the walking speed over 10 meters which was slightly more than in the current study.

However, a lack of training effect or even a slight decrease in walking performance over longer time periods for pwMS has been reported by several authors. In a study by Barrett et al [16] comparing the effects of exercise training and FES for a period of 18 weeks in a group of people with Secondary Progressive MS, the average walking speed over 10 meters decreased from 0.79 m/s to 0.73 m/s in the FES group. However, again, this decrease did not reach statistical significance. The results of Barrett's study agree with earlier work by Taylor et al [11] who also observed a (non statistically significant) decrease in walking speed (0.03 m/s) and increase in Physiological Cost Index (0.13) after four and half months of FES use in pwMS.

Over an even longer time period, Stein et al [9] reported that at the 11 months assessment point, a group of people with progressive diseases (mainly MS) did not show the same degree of continued improvement as seen in a group of people with non-progressive diseases, but nonetheless a small training effect (5.6%) was maintained for 10 m test. The influence of the progressive nature of MS was also highlighted in the results of the study by Taylor et al in 2013 [12]. Unlike those recovering from a stroke, the people with MS in this recent study did not show a mean training effect over a period of 16.5 months. However, 12 out of 35 patients did achieve a meaningful increase in speed of walking defined as more than 0.05 m/s [32] while 10 patients showed a decrease in speed of walking of 0.05 m/s or more.

The finding that some participants showed improvements whilst others deteriorated, resulting in an average neutral effect, was also observed in our study. Although no statistical significant effect of time was found for the 10 meter test, based on the MDC data derived from the results in this study, individual participant responses indicated that after 12 weeks 5 out of the 9 participants showed an improvement in walking speed without FES over 10 meters.

The participants in our study did not show any changes in self-reported walking performance (MSWS-12), impact of MS on daily living (MSIS29), fatigue or objective physical activity after 12 weeks of using FES. Interestingly, there was significant improvement in the MSWS at 6 weeks (average of 6 points), at which point the MSIS29 (average of 9 points) and the daily step count (average of 539 steps) also showed trends towards improvement. However, these trends towards improvement were not maintained at 12 weeks (Fig. 2). A statistically significant improvement in both components of the MSIS29 after 6 weeks of FES was also found in the study by Taylor et al [12] although the improvement in the total score, observed by these authors, was considerably higher (25.6 points) than in the current study. A possible explanation for the difference between our results and that of Taylor et al. are the higher baseline values for the latter study (87.6 vs. 73.0 for the total score). It is possible that people whose function is more affected by MS may subjectively experience more benefit of FES compared to those less affected.

A limitation of pilot studies such as ours and others [13], [14] is the lack of power to detect statistically significant differences for some of the outcome measures. For example, based on our results, future appropriately powered studies would require at least 24 participants in the trial to detect a statistically significant (p<0.05) training effect for dorsiflexion in swing, and 51 participants for a training effect on walking speed over 10 meters, both based on 80% power and paired t-test data. However, it can be argued that for a progressive disease such as MS with a large variability among participants in their disease progression, a frequency analysis using minimally detectable changes may be just as informative as median or mean group changes.

Table 5 showed that although the majority of participants improved their walking performance both over 10 meters and 2 minutes, this could not be explained by a similar number of participants exceeding the MDC for ankle kinematics. Similarly only a few participants reported an improved walking performance, a lower impact of MS, and a decrease in fatigue and none increased their step count more than the MDC. A lack of a relationship between objective walking performance and self-report measures was also observed by Barrett et al [33] who found that an improvement in objective walking performance due to FES to correct dropped foot was not correlated with perceived Quality of Life after 18 weeks of FES use.

There are several possible explanations for these findings. Firstly, the lack of a correlation could be a result of the clinimetric properties of the outcome measures. Gait kinematics are not only influenced by day to day variability of the walking performance of the participant, but also by test retest marker placement errors, even with strict marker placement protocols [34]. Self-reported measures on the other hand may not be responsive enough to detect small changes in perceived walking ability, quality of life or fatigue. Secondly, an improvement in walking performance on the 10 m and 2 minute test may not only result from improved gait kinematics but also due to an increased confidence and reduced mental and physical effort of walking when using FES to correct foot-drop. Finally, it is also possible that walking over a smooth floor in a gait laboratory may have limited ecological validity to translate to every day walking performance, and thus impact on self-report measures. Further studies exploring the impact of FES to treat foot-drop are warranted to further explore these hypotheses.

## Conclusions

To our knowledge this is the first study investigating the medium-to-long-term effects of FES to correct foot-drop on gait kinematics and self-reported walking ability, fatigue and habitual physical activity in pwMS. This pilot study showed that this type of intervention over a 12 week period appears to offer the potential to improve ankle joint kinematics and improve walking speed over 10 meters and 2 minutes FES compared to the baseline assessment without the assistance of FES. However, although these improved outcomes were directionally similar, it is important to note that improvements in gait kinematics and walking performance were not clearly related. Furthermore, the observed improvements in walking speed and gait kinematics, were not reflected in patient reports of walking performance, impact of MS on daily life, fatigue or objectively measured habitual physical activity (as measured by step count). Further studies into more ADL related measures of walking ability such walking for a longer duration or outdoor walking may provide further insight in the relationship between objectively measured walking performance and perceived walking ability, impact of MS in daily living and fatigue.

## Supporting Information

Checklist S1 **TREND checklist.**
(PDF)

Protocol S1 **Protocol of the study as submitted for ethical approval.**
(PDF)

Approval Letter S1 **Letter from the Local Research Ethics committee approving the study.**
(PDF)
