# Supplementary material for: Habitual Functional Electrical Stimulation Therapy Improves Gait Kinematics and Walking Performance, but Not Patient-Reported Functional Outcomes, of People with Multiple Sclerosis who Present with Foot-Drop
Source: PLoS One. 2014 Aug 18;9(8):e103368. doi: 10.1371/journal.pone.0103368 (PMC4136777; doi:10.1371/journal.pone.0103368)
Supplement: Protocol S1 — Protocol of the study as submitted for ethical approval. (PDF) [file pone.0103368.s002.pdf]

A pilot study to assess the effects of using FES or AFO as an assistive mobility device for a period of 12 weeks by people with MS with foot drop

This research will be conducted as two distinct studies. Study one will investigate, in people with Multiple Sclerosis (pwMS), the effects of 12 weeks use of functional electrical stimulation (FES) on walking performance. The second study will investigate, in pwMS, the effects of 12 weeks use of an ankle foot orthosis (AFO) on walking performance. Each study will be conducted as a within-subject repeated measures pre-post comparison trial. Each study will follow the same protocol.

Identification and approach of potential participants

People with MS who are identified by their physiotherapist as eligible candidates for the use of FES or AFO as part of their routine clinical care will be invited to participate in these studies. Participants will be recruited into either study one (FES) or study two (AFO) according to the clinical decision of which assistive device they are best suited to be prescribed. Two recruitment packs have been produced; one for potential FES study participants and one for potential AFO participants. Potential participants will be supplied with an information pack for the relevant study by their physiotherapist. The information pack will include an invitation letter, participant information sheet and a transfer of contact details (TCD) form.

If the patient is interested in finding out more about the study they are required to complete and sign the TCD form to agree to the clinician forwarding their contact details on to the research team at Queen Margaret University. Once the principal investigator (PI) receives the TCD form they will contact the potential participant. The potential participant will have the opportunity to ask questions about the study at this point.

If the potential participant wants to take part in the study, arrangements will be made (including transport if required) for them to visit Queen Margaret University on 4 occasions over a 16 week period. At their first assessment participants will again have the opportunity to ask any further questions before being asked to complete and sign a consent form.

Inclusion/Exclusion Criteria

People with Multiple Sclerosis, aged between 18 and 70 years of age, who are referred to their physiotherapist to received AFO or FES to combat foot drop will be considered for these studies. Participants who cannot walk without a walking stick or walking frame will be excluded from this study.

Patients who are pregnant or breast feeding will be excluded from the study. If participants experience a relapse during their involvement in the study they will be excluded from further participation. Participants who have cardiac or respiratory disease will also be excluded from the study. If the patient is concurrently participating in any other physiological research projects they will not be accepted into this study.

Interventions - Assistive Devices

Function Electrical Stimulation

FES will be administered using the Odstock Dropped Foot Stimulator (ODFS III, Salisbury, Wiltshire, UK). The device will have been fitted to the patient as part of their routine care by their physiotherapist, who is

qualified to fit the stimulator units. Stimulation parameters of the stimulus will be adjusted to suit each participant. The electrode placement and the device settings will be noted and passed to the PI for use in this study.

Ankle Foot Orthosis

The AFO will be prescribed to the participant by their physiotherapist on an individual basis and fitted as part of their routine clinical care. Therefore different AFO's will be included in the study. However all AFO's will perform the intended function of reducing foot drop.

Timescale

Participants will be involved in this study for 16 weeks during which time we will request that they make 4 visits to the gait lab at Queen Margaret University. Each testing session will take approximately 2 hours to complete.

Protocol

Participants will attend their first assessment before they receive their assistive device. During their first assessment they will only be tested walking without assistance. Once they receive their appropriately prescribed assistive device they will be tested again (with and without their device). Participant will use the device habitually on a day to day basis to assist their walking. They will then be tested on a further two occasions separated by a period of 6 weeks.

Each participant will be tested at the same time of day (+/- 1 hour) each time they are tested.

Testing Sessions

Participants will be asked to report to the main reception at Queen Margaret University and ask for the PI. Participants will be asked to wait at the main reception until a member of the research team collect them and take them to the gait lab in a wheelchair.

Prior to each testing session the computerised 3 dimensional motion analysis system (Vicon) will be calibrated using a standardised method according to the Vicon manual.

The study will be fully explained to the participant at the start of the assessment session 1. They will be shown the equipment and the laboratory and have the opportunity to ask questions. Once all questions have been satisfied and the participant is happy to proceed they will be asked to complete and sign a consent form before commencing testing.

Participants will carry out eight 6-7-metre walks without their assistive device and eight 6-7-metre walks with their assistive device while their gait is digitally recorded using Vicon three dimensional motion analysis (Vicon, Nexus, Oxford, UK). Participants will be asked to walk how they would do normally at their preferred walking speed.

Participants will also be asked to carry out one timed 10 metre walk without their assistive device and one timed 10 metre walk with their assistive device (Paltamaa *et al.*, 2005). In addition participants will be asked to carry out two 2-minute walk tests, one with and one without their assistive device.

At each testing time point participants will be asked to complete four short questionnaires. They will also be given an activpal physical activity monitor to take away with them at the end of each testing session to wear for a week.

Procedures

Physical Measurements

Anthropometric and information (height and weight, knee width, ankle width and leg length) will be collected at each visit for the calculation of the ankle, knee and hip joint centres by the motion analysis software ('Plug in Gait').

Body composition assessment by multi-freqency, bioelectrical impedance analysis (BIA) will also be be carried out at the beginning of each testing session using a Maltron Bioscan 920-2. While the participant is lying down at rest we attach electrodes to their foot, ankle, hip, shoulder, wrist and hand (on their left hand side). An electrical impulse is passed between the electrodes (which the participant does not feel). The BIA analyser records the time taken for the electrical impulse to travel between each electrode and uses these results to calculate body composition.

Gait Analysis

Participants will have 14mm reflective sphere makers, which are visible to the infra red cameras, placed on their lower limbs and pelvis, using skin friendly hypoallergenic double sided tape. They will also be asked to wear black cycling shorts (provided) so that markers can be attached directly to their hips.

10m Walking Test

Participants will be asked to walk in a straight line at their preferred walking speed (PWS) through the start line, straight towards the end of the walkway. The time to complete the 10m walking test will be recorded manually using a stop watch. The clock will be started and stopped when a foot either falls on or passes over the start and finish line respectively. Seconds will be recorded to two decimal places.

2 Minute Walking Test

Two 2-minute walk tests will be conducted at each testing session. Participants will be given a 15 minute rest in between the two 2-minute walks to ensure they start both walks in the same physiologically rested state. Participants will be asked to walk continuously around a 16m elliptical course for 2 minutes at their preferred walking speed. The distance travelled will be recorded by recording split times each time they complete a lap and by marking the point on the course they reach in 2 minutes. If they need to stop they can stand or lean against a wall and then continue. If they need to stop and sit down the test will be terminated.

Participants will be asked to rate their perceived exertion (RPE), according to the Borg category rating scale (Borg, 1970) in the middle and at the end of 2-minute walk test.

Participants will be asked to mark on a 10cm line labelled from 'no fatigue' to 'severe fatigue' (Johnson, 2005), how fatigued they feel immediately before and after the 2 minute walk test.

Questionnaires

Participants will be asked to complete the following four questionnaires at each assessment. After the first assessment the four questionnaires will be posted out to the participants a week prior to their next assessment. They will be asked to complete the questionnaires again and send them back to Queen Margaret University in a pre paid envelope or bring them in with them to the next assessment.

Fatigue will be assessed by:

- Fatigue Severity Scale (Krupp *et al.*, 1989)

Activity of Daily Living will be assessed by the:

- 12 Item MS Walking Scale (Hobart *et al.*, 2003)

Quality of Life will be assessed using:

- Leeds MS Quality of Life Questionnaire (Ford *et al.*, 2001)
- MS Impact Score 29 (Hobart *et al.*, 2001)

Physical Activity Monitoring

Physical activity levels will be measured using the activpal™ activity monitor. This is a small accelerometer device (size 53 x 35 x 7 mm; weight 20g) which attaches directly to the skin of the front of the thigh using activpal adhesive stickers (activstickies).

Participants will be asked to wear the device 24 hours a day for 7 days after each testing sessions.

Statistics

Sample Size Calculation

Both the FES and the AFO studies are pilot studies and with the aim being to help determine the factors which will inform the sample size (e.g. variance) for the main study and to assess the feasibility of steps that need to be taken for a future study such as recruitment and retention rates (Thabane *et al.*, 2010).

A sample size of 6 will have 80% power to detect an effect size of 1.435 using a paired t-test with a 0.050 two-sided significance level. Taking into account data loss and attrition this was inflated to 12 participants.

Analysis of Data

Following checks for normality the gait kinematic data will be analysed using paired t-tests. Where appropriate; parametric tests will be used to evaluate the effect of the respective interventions on the primary outcome measure of gait kinematics. A similar statistical approach will be applied to the times walking performance and the physical activity data. If parametric assumptions are not met appropriate non parametric tests (Wilcoxon test) will be employed.

In all analyses a significance level of $p<0.05$ will be regarded as statistically significant.

Ethical Opinion

This study has been reviewed and approved by the NHS Lothian ethics and management committee. In addition the Queen Margaret University research ethics committee have approved this study.

Reference List

Borg G (1970). Perceived exertion as an indicator of somatic stress. *Scandinavian journal of rehabilitation medicine* **2**, 92-98.

Ford HL, Gerry E, Tennant A, Whalley D, Haigh R, & Johnson MH (2001). Developing a disease-specific quality of life measure for people with multiple sclerosis. *CLIN REHABIL* **15**, 247-258.

Hobart J, Lamping DL, Fitzpatrick R, Riazi P, & Thompson A (2001). The Multiple Sclerosis Impact Scale (MSIS-29): a new patient based outcome measure. *Brain* **124**, 962-973.

Hobart JC, Riazi A, Lamping DL, Fitzpatrick R, & Thompson AJ (2003). Measuring the impact of MS on walking ability: The 12-Item MS Walking Scale (MSWS-12). *Neurology* **60**, 31-36.

Johnson C (2005). Measuring Pain. Visual Analogue Scale versus Numeric Pain Scale: What is the Difference? *Journal of Chiropractic Medicine* **4**, 43-44.

Krupp LB, LaRocca NG, Muir-Nash J, & Steinberg AD (1989). The Fatigue Severity Scale: application to patients with multiple sclerosis and systemic lupus erythematosus. *Archives of Neurology* **46**, 1121-1123.

Paltamaa J, West H, Sarasoja T, Wikstrom J, & Milkia E (2005). Reliability of physical functioning measures in ambulatory subjects with MS. *Physiotherapy Research International: The Journal For Researchers And Clinicians In Physical Therapy* **10**, 93-109.

Thabane L, Ma J, Chu R, Cheng J, Ismaila A, Rios LP, Robson R, Thabane M, Giangregorio L, & Goldsmith CH (2010). A tutorial on pilot studies: the what, why and how. *BMC Medical Research Methodology* **10**, 1.
