# Supplementary material for: Habitual Functional Electrical Stimulation Therapy Improves Gait Kinematics and Walking Performance, but Not Patient-Reported Functional Outcomes, of People with Multiple Sclerosis who Present with Foot-Drop
Source: PLoS One. 2014 Aug 18;9(8):e103368. doi: 10.1371/journal.pone.0103368 (PMC4136777; doi:10.1371/journal.pone.0103368)
Supplement: Approval Letter S1 — Letter from the Local Research Ethics committee approving the study. (PDF) [file pone.0103368.s003.pdf]

Lothian NHS Board

South East Scotland Research Ethics Committees
Waverley Gate
2-4 Waterloo Place
Edinburgh
EH1 3EG
www.nhslothian.scot.nhs.uk

NHS Lothian

01 June 2011

Enquiries Emily Pendleton
Direct Line 0131 465 5676
emily.pendleton@nhslothian.scot.nhs.u

Miss Sasha Scott
Postgraduate Research Student
Queen Margaret Univeristy
Queen Margaret University
Musselburgh
East Lothian
EH21 6UU

Dear Miss Scott

Study title: **A pilot study to assess the effects of using FES or AFO as an assistive mobility device for a period of 12 weeks by people with MS with foot drop.**
REC reference: **11/AL/0229**

Thank you for your letter of 24 May 2011, responding to the Committee's request for further information on the above research and submitting revised documentation.

The further information was considered in correspondence by a sub-committee of the REC at a meeting held on 1 June 2011. A list of the sub-committee members is attached.

Confirmation of ethical opinion

On behalf of the Committee, I am pleased to confirm a favourable ethical opinion for the above research on the basis described in the application form, protocol and supporting documentation as revised, subject to the conditions specified below.

Ethical review of research sites

NHS sites

The favourable opinion applies to all NHS sites taking part in the study, subject to management permission being obtained from the NHS/HSC R&D office prior to the start of the study (see "Conditions of the favourable opinion" below).

Non-NHS sites

Conditions of the favourable opinion

NHS Lothian

The favourable opinion is subject to the following conditions being met prior to the start of the study.

Management permission or approval must be obtained from each host organisation prior to the start of the study at the site concerned.

Management permission ("R&D approval") should be sought from all NHS organisations involved in the study in accordance with NHS research governance arrangements.

Guidance on applying for NHS permission for research is available in the Integrated Research Application System or at http://www.rdforum.nhs.uk.

Where a NHS organisation's role in the study is limited to identifying and referring potential participants to research sites ("participant identification centre"), guidance should be sought from the R&D office on the information it requires to give permission for this activity.

For non-NHS sites, site management permission should be obtained in accordance with the procedures of the relevant host organisation.

Sponsors are not required to notify the Committee of approvals from host organisations

It is the responsibility of the sponsor to ensure that all the conditions are complied with before the start of the study or its initiation at a particular site (as applicable).

Approved documents

The final list of documents reviewed and approved by the Committee is as follows:

Document	Version	Date
Covering Letter		31 March 2011
Evidence of insurance or indemnity		
GP/Consultant Information Sheets	1	24 May 2011
Investigator CV		
Letter from Sponsor		
Letter from Statistician		
Letter of invitation to participant		31 March 2011
Other: Visual Analogue Scale	1	31 March 2011
Other: RPE Scale	1	31 March 2011
Other: funder letter		
Other: Invitation	1	31 March 2011

Other: FES	1	24 May 2011
Participant Consent Form: AFO	2	24 May 2011
Participant Consent Form: FES	2	24 May 2011
Participant Information Sheet: FES	2	24 May 2011
Participant Information Sheet: AFO	2	24 May 2011
Protocol	1	31 March 2011
Questionnaire: Life Questionnaire		
Questionnaire: FSS		
Questionnaire: MS Impact Scale		
Questionnaire: MS Walking Scale		
REC application		06 April 2011
Response to Request for Further Information		
Summary/Synopsis	1	31 March 2011

Statement of compliance

The Committee is constituted in accordance with the Governance Arrangements for Research Ethics Committees (July 2001) and complies fully with the Standard Operating Procedures for Research Ethics Committees in the UK.

After ethical review

Now that you have completed the application process please visit the National Research Ethics Service website > After Review

You are invited to give your view of the service that you have received from the National Research Ethics Service and the application procedure. If you wish to make your views known please use the feedback form available on the website.

The attached document “After ethical review – guidance for researchers” gives detailed guidance on reporting requirements for studies with a favourable opinion, including:

- Notifying substantial amendments
- Adding new sites and investigators
- Progress and safety reports
- Notifying the end of the study

The NRES website also provides guidance on these topics, which is updated in the light of changes in reporting requirements or procedures.

We would also like to inform you that we consult regularly with stakeholders to improve our service. If you would like to join our Reference Group please email referencegroup@nres.npsa.nhs.uk.

11/AL/0229	Please quote this number on all correspondence

With the Committee's best wishes for the success of this project

Yours sincerely

Dr Janet Andrews
Chair

Email: emily.pendleton@nhslothian.scot.nhs.uk

Enclosures:	List of names and professions of members who were present at the meeting and those who submitted written comments<br><br>"After ethical review – guidance for researchers"
Copy to:	Dr Fiona Coutts<br>N/A. R&D contact not specified in database.

NHS Lothian

South East Scotland Research Ethics Committee 01

Attendance at Sub-Committee of the REC meeting on 1 June 2011

Committee Members:

Name	*Profession*	*Present*	*Notes*
Dr Janet Andrews	Associate Specialist	Yes	
